# Quantitative dual-energy micro-CT with a photon-counting detector for material science and non-destructive testing

**DOI:** 10.1371/journal.pone.0219659

**Published:** 2019-07-17

**Authors:** Thorsten Sellerer, Sebastian Ehn, Korbinian Mechlem, Manuela Duda, Michael Epple, Peter B. Noël, Franz Pfeiffer

**Affiliations:** 1 Chair of Biomedical Physics, Department of Physics and Munich School of Bioengineering, Technical University of Munich, Garching, Germany; 2 Department of Diagnostic and Interventional Radiology, Technical University of Munich, Klinikum Rechts der Isar, München, Germany; 3 MITOS GmbH, Garching, Germany; 4 Institute for Advanced Study, Technical University of Munich, Garching, Germany; University of Notre Dame, UNITED STATES

## Abstract

The recent progress in photon-counting detector technology using high-Z semiconductor sensors provides new possibilities for spectral x-ray imaging. The benefits of the approach to extract spectral information directly from measurements in the projection domain are very advantageous for material science studies with x-rays as polychromatic artifacts like beam-hardening are handled properly. Since related methods require accurate knowledge of all energy-dependent system parameters, we utilize an adapted semi-empirical model, which relies on a simple calibration procedure. The method enables a projection-based decomposition of photon-counting raw-data into basis material projections. The objective of this paper is to investigate the method’s performance applied to x-ray micro-CT with special focus on applications in material science and non-destructive testing. Projection-based dual-energy micro-CT is shown to be of good quantitative accuracy regarding material properties such as electron densities and effective atomic numbers. Furthermore, we show that the proposed approach strongly reduces beam-hardening artifacts and improves image contrast at constant measurement time.

## Introduction

The concept of hybrid-pixel photon-counting detectors (PCDs) has been demonstrated for the first time at CERN in 1991 [1, 2] for the application as particle tracking detectors in high-energy physics. Since then, due to the benefits this technology implies, a lot of effort has been made to investigate the applicability of PCDs in x-ray imaging. In contrast to state-of-the-art integrating detectors, each photon is registered as a single event and is not affected by energy weighting common for flat-panel detectors. Therefore, PCDs can deliver an intrinsically higher image contrast [3]. The detection of photons is realized by discriminators which register a photon only if its energy exceeds a certain threshold (THL) value. This detection approach guarantees that electronic noise is eliminated effectively and consequently these type of detector does not suffer from dark current signals. The implementation of additional discriminators to the detector allows setting different threshold levels resulting in a discrimination of a polychromatic x-ray spectrum into several distinct energy bins.

The significant progress of PCD technology achieved over the last years [3–5] created a promising alternative to common energy-integrating detector systems predominant in x-ray imaging nowadays. Especially the ability of PCDs to handle a high photon flux as well as ensuring a sufficient quantum efficiency by the use of high-Z semiconductor sensor materials makes them a viable alternative for state-of-the-art imaging applications.

The concept of spectral x-ray imaging has first been developed for clinical CT in the late 1970’s by Alvarez and Macovski [6]. For the most general case in spectral x-ray imaging we consider a CT scan of an object composed of a number *K* of materials at *N* ≥ *K* energy levels *E*_1_⋯*E*_*N*_. The obtained set of energy-dependent CT images *μ*(*E*_*i*_) can be expressed by the vector

$$\begin{array}{c} \left(\begin{array}{c} \mu \left(E_{1}\right)\\ \vdots \\ \mu \left(E_{N}\right) \end{array}\right)\;=\;\underset{B}{\underset{︸}{\left(\begin{array}{ccc} f_{1}\left(E_{1}\right) & \cdots & f_{K}\left(E_{1}\right)\\ \vdots & \ddots & \vdots \\ f_{1}\left(E_{N}\right) & \cdots & f_{K}\left(E_{N}\right) \end{array}\right)}}\;\left(\begin{array}{c} a_{1}\\ \vdots \\ a_{K} \end{array}\right), \end{array}$$ (1)

with the *N* × *K* spectral basis function matrix *B*. The process of retrieving the basis material images *a*_1_ … *a*_*K*_ from the measured spectral attenuation data is called material decomposition. Significant benefits of these spectral imaging methods using x-rays have already been demonstrated for many clinical applications [7–10]. However, the capability of measuring the material-specific attenuation of x-rays with respect to its energy dependency also introduces new possibilities to the field of material science. As an example, the decomposition of the total attenuation into spectral basis materials enables the possibility to create virtual mono-energetic images (VMI) without beam-hardening artifacts. Furthermore, underlying physical and chemical material properties such as atomic numbers or electron densities can be quantified. To this time, there exist several methods and algorithms to exploit the obtained spectral data, which can in principle be classified into projection- and image-based methods.

Image-based approaches rely on reconstructed polychromatic CT images obtained with different effective x-ray spectra and therefore perform the material decomposition in the image domain, i.e. such methods perform the decomposition after the CT reconstruction. The most basic implementation uses a 2D scatter-plot of the voxel attenuation values at low and high energies, respectively. Using vector decomposition, some information about the sample composition can be obtained. Furthermore, previously known values of the energy-dependent spectral basis functions obtained in dedicated calibration scans can be used to directly invert the system of Eq (1). More advanced approaches uses linear combinations of the reconstructed images and allow to incorporate additional constraints on the material composition of the sample [11, 12]. Image-based methods generally have the disadvantage that beam-hardening artifacts are not accounted for correctly and need to be treated separately [13].

The second concept consists of raw data-based methods that performs the decomposition directly in the projection domain prior to CT image reconstruction, yielding basis component line-integrals. Methods that rely on a basis material decomposition in the projection domain are by their design capable of correctly modeling the hardening of polychromatic x-ray spectra penetrating an object. Hence, this concept is a very promising tool for quantitative x-ray imaging of material-science related samples since metal artifacts are often a major issue. In first attempts, the projection data obtained in dual-energy acquisitions was modeled by polynomials considering the energy dependence of Compton scattering and photoelectric absorption as the two predominant physical causes of attenuation [6]. More recent concepts are capable of handling more than two energy bins and expanding the amount of basis materials to K-edge discontinuities [14, 15]. However, these approaches require extensive amounts of characterization and calibration measurements that can be very challenging to implement in typical imaging set-ups.

Therefore, a new concept based on a semi-empirical polychromatic Beer-Lambert forward model has been developed by the authors featuring a strongly simplified and fast calibration routine. In prior simulations [16], this method was shown to yield decomposed data with statistical minimum in variance (Cramér-Rao lower bound [14] and reference in there) and being unbiased.

The previously published work only used simulated data and therefore neglected some effects that are incorporated when using PCDs in an experimental environment. Among those effects the most influential in an experimental measurement are sensor polarization [17], threshold drift [18] and interpixel variations of the detector response. The following work aims at evaluating the proposed approach in terms of the achievable quantitative accuracy in experimental measurements and its practicalness in material-science studies and non-destructive-testing (NDT). The imaging setup used allows for a dual energy approach, enabling a basis material decomposition in two materials. A special focus of the performed studies was on the determination of electron density (*ρ*_el_) and effective atomic number (*Z*_eff_) values within unkown objects. Although, there is a lot of literature on the determination of those quantities from dual-energy measurements [19–21], most of the work published so far focuses on tissue-like materials exhibiting rather low electron density and effective atomic number values. However, in NDT applications the sample of interest often contains high-Z materials such as metals, in which case an accurate determination of the quantities *ρ*_el_ and *Z*_eff_ is challenging especially when using image-based approaches. Beam-hardening is a prominent effect occurring in conventional CT, especially when strongly absorbing structures like metals are present in the studied object. Thereby, cupping and streaks in the reconstructed images often decrease the quality and complicate the analysis and interpretation of CT images investigated for NDT [22].

The paper is structured in the following order. After recapitulation of the proposed model and giving a brief illustration of the algorithm used for the decomposition in the methods section, a detailed description of the calibration procedure and the used experimental set-up will be given. In the results section the quantitative accuracy and quality of the acquired spectral images will be illustrated based on phantom measurements. Furthermore, the method was used for imaging of a concrete drill core and a ethernet connector to study its practicalness in NDT applications. Subsequently, we will discuss the obtained results.

## 1 Materials and methods

### Determination of the basis material line-integrals and image reconstruction

In this study we make use of maximum-likelihood estimation (MLE) [23] of the basis material line-integrals relying on a semi-empirical estimator of the signals measured by an energy-resolving photon-counting detector. The key aspects of this approach are discussed in detail in [16] and [24], and therefore will only be briefly outlined in the following section.

The linear attenuation coefficient of any material without K-edge discontinuities in the respective energy range (20 keV − 150 keV), can be approximated by a linear combination of two linearly independent contributions, the so-called basis materials [6, 25]:

$$\begin{array}{c} \mu \left(\vec{x},\;E\right)≃a_{1}\left(\vec{x}\right)\cdot \mu_{\text{1}}\left(E\right)+a_{2}\left(\vec{x}\right)\cdot \mu_{\text{2}}\left(E\right), \end{array}$$ (2)

where *μ*_1,2_ are the attenuation coefficients of the basis materials and *a*_1,2_ scalar factors describing their respective contribution to the total attenuation. The vector $\vec{x}$ labels the position of the respective voxel in the image volume.

In projection space, the number of photon counts *C*_*s*_ registered in energy bin *s* of a photon-counting detector is a function of the basis material line-integrals:

$$\begin{array}{c} A_{\text{1}}=\int_{0}^{S}\;a_{\text{1}}\left(\vec{x}\right)\text{d}s,\;\;\;A_{\text{2}}=\int_{0}^{S}\;a_{\text{2}}\left(\vec{x}\right)\text{d}s\;\;\;. \end{array}$$ (3)

In practice, an accurate calculation of *C*_*s*_ would require exact knowledge of energy-dependent set-up parameters such as the x-ray spectrum emitted by the tube source and the specific energy response of the utilized detector [15, 26].

An accurate experimental determination of these quantities, especially concerning the bin sensitivity, is a huge effort. Furthermore, the required parameters alter over the lifetime of the detector and the x-ray source. Adding to that, the specific position of thresholds and the spectral response function of any real PCD slightly deviates from pixel to pixel. Small fluctuations of these parameters can be caused by the environment, such as variations in temperature and periodic re-calibration on a pixel-individual basis is required consequently. In the light of this, a semi-empirical approach based on the physical attenuation properties but relying on a simple calibration routine has been developed to extract spectral data from measurements [16, 24]. The proposed model gives the expected counts registered by the detector in each energy bin *s* and pixel *j* by

$$\begin{array}{c} C_{sj}=\sum_{m=1}^{M}C_{sj}^{m}\cdot e^{-(A_{\text{1}}\mu_{\text{1}}\left({\hat{E}}_{sj}^{m}\right)\;+\;A_{\text{2}}\mu_{\text{2}}\left({\hat{E}}_{sj}^{m}\right))}\;, \end{array}$$ (4)

where the ${\hat{E}}_{sj}^{m}$ and $C_{sj}^{m}$ are fit parameters describing the attenuation caused by the line-integrals *A*_1_ and *A*_2_. Thereby, $\sum_{m=1}^{M}C_{sj}^{m}$ preserves the incident photon flux registered by the detector in energy bin *s* and detector pixel *j*. Effectively, the polychromatic x-ray spectrum is replaced by a small number of distinct energy levels ${\hat{E}}_{sj}$ serving as a surrogate spectrum to describe the energy dependent attenuation caused by the basis materials. The number of required terms *M* can vary depending of the material thickness penetrated by the beam or rather the amount of beam-hardening introduced. The model parameters are defined pixelwise and hence allow for proper consideration of variations between the individual pixel’s spectral response.

In order to perform a material decomposition on a projection dataset, Eq 4 has to be numerically solved for the line-integrals *A*_1_ and *A*_2_ considering the count data registered in the different energy bins. This can be accomplished by performing a MLE, i.e. minimizing the log-likelihood function $L({\hat{c}}_{sj},\;C_{sj})$ for given measured photon counts ${\hat{c}}_{sj}$ and basis material line-integrals.

This process yields the MLE results for *A*_1_ and *A*_2_ referred to as *A*_ML_ by

$$\begin{array}{c} A_{\text{ML}}=\underset{A_{\text{1}},A_{\text{2}}}{\text{argmin}}\;\;L\left({\hat{c}}_{sj},\;A_{\text{1}},\;A_{\text{2}}\right). \end{array}$$ (5)

The exact construction and mathematical properties of the cost-function $L({\hat{c}}_{sj},\;C_{sj})$ is extensively discussed in the available literature [16, 26]. Most notably, MLE processing of photon-counting data can be shown to be an unbiased and efficient, i. e. minimum noise, solution to the spectral reconstruction problem [16, 27, 28].

The reconstruction of CT scans performed in this study was done with an in-house developed tomographic projector [29], yielding the basis material voxel volume fractions $a_{\text{1}}\left(\vec{x}\right)$ and $a_{\text{2}}\left(\vec{x}\right)$ of the studied object. The availability of basis material images allows to calculate virtual monoenergetic images (VMI) for a selectable photon energy *E* = *E*_0_ using Eq 2:

$$\begin{array}{c} \mu \left(E_{0},\vec{x}\right)≃a_{\text{1}}\left(\vec{x}\right)\cdot \mu_{\text{1}}\left(E_{0}\right)+a_{\text{2}}\left(\vec{x}\right)\cdot \mu_{2}\left(E_{0}\right). \end{array}$$ (6)

Using the basis material volume fractions, the chemical properties of a studied object can be extracted in form of the effective electron density and effective atomic number.

For a known composition of a material, the electron density can be calculated by

$$\begin{array}{c} \rho_{\text{el}}=\rho \cdot \frac{\sum p_{i}Z_{i}}{\sum p_{i}A_{i}}\cdot \frac{1}{1\;\text{u}}, \end{array}$$ (7)

where *ρ* is the mass density of the material and 1 u is 1 atomic mass unit. The weights *p*_*i*_ account for the number fraction of atom *i* with the atomic number *Z*_*i*_ and the atomic mass number *A*_*i*_.

According to [30] the effective atomic number of a material can be calculated by

$$\begin{array}{c} Z_{\text{eff}}=\sqrt[{3.0}]{\sum w_{i}\cdot Z_{i}^{3.0}}, \end{array}$$ (8)

with *w*_*i*_ being the fractional mass contributed to a mixture by element *i* with atomic number *Z*_*i*_.

As the basis material volume fractions represent the attenuation properties of an investigated object, they also serve as a surrogate quantity for the studied object’s electron density and atomic number, which are determining for a material’s attenuation characteristics.

To calculate the electron density within an image voxel of the reconstructed CT data, we weight the obtained basis material volume fractions with the electron densities of the known basis materials:

$$\rho_{\text{el},\text{eff}}(\vec{x})=a_{\text{1}}(\vec{x})\cdot \rho_{\text{el},\text{1}}+a_{\text{2}}(\vec{x})\cdot \rho_{\text{el},\text{2}},$$ (9)

where *ρ*_el,1,2_ are the electron densities of basis material 1 and 2, respectively.

The effective atomic number within an image voxel is calculated according to Eq 8, where the fractional masses of the basis materials are used instead of the true composition of the studied object:

$$\begin{array}{c} Z_{\text{eff}}\left(\vec{x}\right)=\sqrt[{3.0}]{w_{1}\left(\vec{x}\right)\cdot Z_{1}^{3.0}+w_{2}\left(\vec{x}\right)\cdot Z_{2}^{3.0}}. \end{array}$$ (10)

Thereby, *w*_1_, *w*_2_ are the mass fractions of basis material 1,2 within the voxel and *Z*_1_, *Z*_2_ are the atomic numbers of the basis materials. The mass fractions in the corresponding voxel can be calculated by:

$$\begin{array}{c} w_{i}=\frac{a_{i}\left(\vec{x}\right)\cdot \rho_{i}}{a_{1}\left(\vec{x}\right)\cdot \rho_{1}+a_{2}\left(\vec{x}\right)\cdot \rho_{2}}, \end{array}$$ (11)

with *ρ*_1,2_ labeling the mass densities of the basis materials.

### 1.1 Calibration of system parameters

The parameters in Eq 4 contain the information about the whole imaging device, including the properties of the source spectrum as well as the detector response. In order to determine the model parameters, a calibration routine is used which relies on the measurement of different line-integral combinations of two well-known materials, yielding a set of calibration points

$$\begin{array}{c} A^{p,q}=\left(A_{1}^{p},A_{2}^{q}\right), \end{array}$$ (12)

where the $A_{1}^{p}$ and $A_{2}^{q}$ are the thicknesses of the materials used in the calibration routine. The energy depedendencies (*μ*_1_(*E*), *μ*_2_(*E*)) in Eq 4 are the attenuation coefficients of the used calibration materials and can be found in the XCOM database [31].

Due to its reliability and unbiasedness [27], a MLE method is again used to calibrate the system parameters $\left(C_{s}^{0},\ldots ,C_{s}^{M};{\hat{E}}_{s}^{0},\ldots ,{\hat{E}}_{s}^{M}\right)$ in Eq 4 analogous to the approach explained above but assuming known values for *A*_1_ and *A*_2_ for the calibration. The number of exponential terms in Eq 4 used for our experiments was M = 4.

### 1.2 Optimization of threshold positions and noise reduction

As common for spectral imaging techniques, the Poisson noise present in the registered count data propagates into the estimates of the basis materials. Whereas there is no correlation between the data in the different energy bins, the estimation introduces an anti-correlation to the basis material line-integrals, which also affects the image noise [27, 32]. The lower limit for the variance in the basis material images is given by the Cramér-Rao lower bound (CRLB) [14], which can be utilized to optimize the spectral binning of the registered count data to minimize the noise present in the basis material images according to the method presented in [33]. After decomposition of the acquired energy-binned raw data into basis materials according to Eq 5, the projections were reconstructed by FBP using a Ram-Lak filter [34] to preserve spatial resolution. Besides this approach of noise reduction by a-priori optimization of acquisition parameters, there are several sophisticated de-noising algorithms, which proved to perform well in handling anti-correlated noise. For our studies we used an in-house developed dictionary-based algorithm [35]. A conventional, polychromatic CT image was also obtained for each scan by summing up the contributions of all energy bins. The conventional images have also been post-processed by the above mentioned dictionary-based algorithm. However, in this case the algorithm was only applied to a single volume. To enable a fair comparison between the imaging modalities, the smoothing strength parameters of the algorithm were choosen for a similar visual appearance in terms of image resolution, respectively.

### 1.3 Experimental setup

The experimental measurements were performed on a tomographic setup consisting of a micro-focus x-ray tube, a photon-counting detector system and several positioning devices mounted on an optical table.

The statically mounted x-ray tube (XWT-160-CT, X-RAY WorX GmbH, Garbsen, Germany) is a micro-focus tube with a tungsten reflection target and a 2mm thick beryllium window. It can be operated at tube voltages of up to 160 kVp and has a maximum target power output of 300 W. Furthermore, the tube has a minimal focal spot size of 2.0/3.0 *μ*m(hor./vert.), which broadens with increasing target power to its maximum of approximately 150/320 m at a power of 120 W [36]. The beam has an opening angle of about 30° and can be varied in its shape by an additionally attached collimator. The used detector system (Flite X1, Direct Conversion AB, Danderyd, Sweden) is a photon-counting hybrid pixel detector with a 750 m Cadmium-telluride (CdTe) sensor. The detector’s active area is 1536 x 128 pixels (≈ 155 x 13mm^2^) with a native pixel size of 100 m, arranged in a horizontal pattern of 12 chip modules with a chip gap of one pixel. The system has two energy thresholds per pixel and comes with an integrated charge sharing correction. The maximum acquisition rate is up to 200 ⋅ 10^6^ $\frac{\text{photons}}{\text{s}\cdot {\text{mm}}^{2}}$.

**Fig 1:**
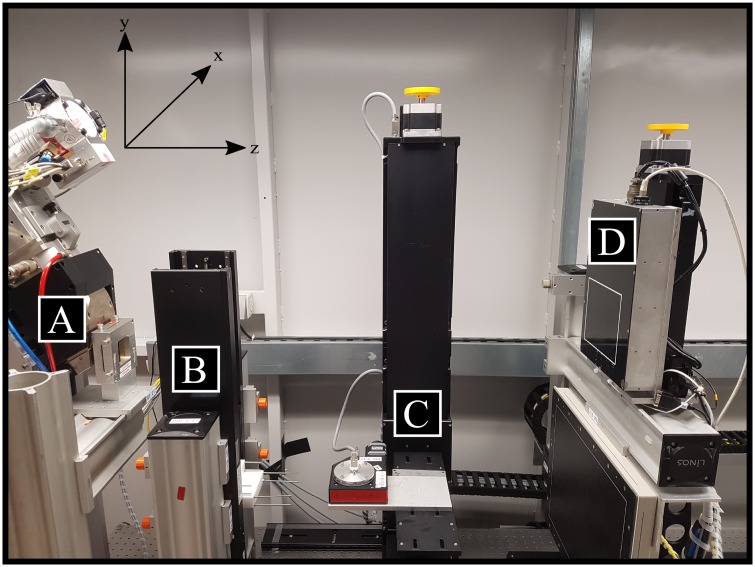
Experimental setup. Photography of the spectral micro-CT set-up: (A) micro-focus x-ray source with beam collimator, (B) linear-stages with calibration phantoms to automatically calibrate the model parameters, (C) mounting and positioning devices for the sample and adjustment of magnification and voxel-size, (D) photon-counting detector.

The set-up is equipped with a sample holder completely movable in *x*-, *y*- and *z*-direction, due to an arrangement of three linear stages. The rotation of the sample is realized by an additional rotation stage. The detector system can further be moved in the z-, and y-direction. Thus the set-up allows a flexible and accurate geometric alignment, including arbitrary selection of the geometric magnification as well as the correct alignment of the sample and the detector to the center of the cone beam. Fig 1 shows a photograph of the described components.

The calibration phantoms are mounted onto two additional linear-stages between the x-ray tube and the sample holder. The phantoms consist of an aluminum frame that is equipped with cuboid blocks of Polyoxymethylen (POM, CH2O) and titanium foils (purity > 99.7%).

## 2 Results

### 2.1 Calibration reliability

**Fig 2:**
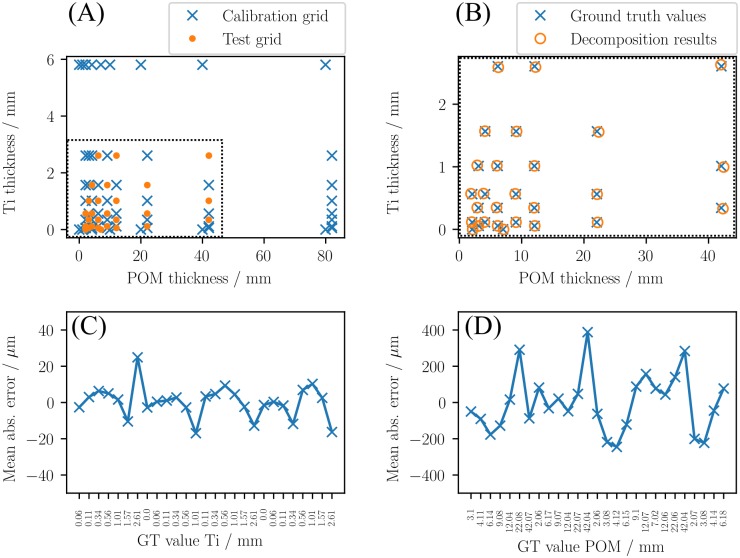
Decomposition accuracy. In order to asses the reliability of the calibration of the forward-model in Eq 4 a testgrid different from the calibration grid was acquired (A). The measured dual-energy data was decomposed into basis material thicknesses (B) and compared to the ground truth (GT) values for Ti (C) and POM (D), respectively.

In order to assess the performance of the calibration routine a test grid of various thickness combinations of titanium and POM was consulted. The parameters in Eq 4 were determined by the described MLE method after performing a calibration routine with the calibration grid indicated by the blue crosses in Fig 2A. The energy thresholds were set to 23 and 64 keV (CRLB optimization), while the source peak voltage was 120 kVp. The photon statistic was kept constant for all thickness combinations by adjusting the exposure time (≈ 2.5 ⋅ 10^4^ counts per pixel).

After the calibration routine the test-grid data was acquired and decomposed into line-integrals of titanium and POM. The test grid was different from the calibration grid and is indicated by the orange dots in Fig 2A.

Fig 2B shows the resultant line integral values (orange circles) alongside with the respective ground truth values (blue crosses) within the evaluated line-integral region (dotted rectangle). The relative bias between the decomposed and true thickness values was calculated by

$$\begin{array}{c} \Delta A^{k,l}=\sum_{j=0}^{J}\frac{A_{j}^{k,l}-{\hat{A}}^{k,l}}{{\hat{A}}^{k,l}}, \end{array}$$ (13)

where the $A_{j}^{k,l}={\left(A_{1}^{k},A_{2}^{l}\right)}_{j}$ give the decomposed line-integral values in detector pixel *j*, and ${\hat{A}}^{k,l}$ are the respective ground truth values. Thereby, the *k* and *l* label the titanium and POM thicknesses of the evaluated ‘test-grid’ in Fig 2A.

The total number of evaluated pixels was *J* = 40 × 1032, corresponding to the collimated detector area during the measurement. Fig 2C and 2D show the resultant deviations, which are in the range of 30 μm for titanium and 400 μm for POM.

### 2.2 Quantitative accuracy in a phantom measurement

Based on a cylindrical phantom featuring various materials with exactly known material properties the quantitative accuracy of the proposed method was investigated. The phantom is made of four cylindrical rods consisting of titanium (Ti), polyacetal (POM, CH2O), aluminum (Al) and teflon (PTFE, C2F4), respectively. The acquisition parameters used for the phantom are summarized in Table 1.

**Table 1:** CT geometry and image parameters of the performed tomographic scans. The field-of-view (FOV) and the pixel-size are given at the isocenter. Projections per 360° and the tube loading are given for the different samples: rod phantom / concrete drill core/ ethernet connector.

FOV	SDD^1^	SSD^2^	pixel-size	projections per 360°
3.0 cm	150 cm	30 cm	20 μm	1600/1600/1400
Anode	Peak Energy	Filter	Thresholds	Tube loading
W	120 kVp	N.A.	23, 64 keV	2000/2000/1750 mAs

^1^ Source-to-detector distance

^2^ Source-to-sample distance

**Fig 3:**
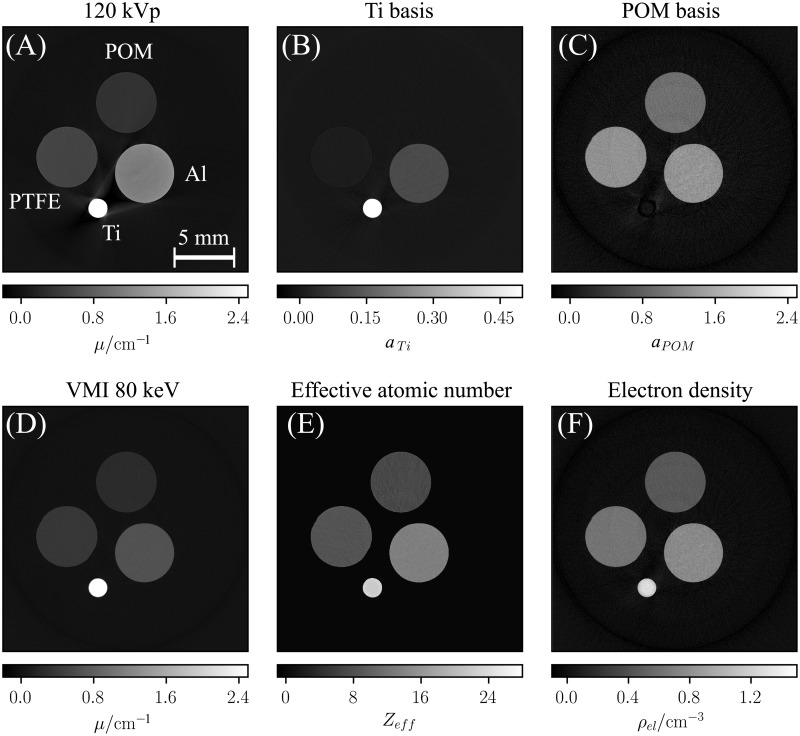
Spectral CT images of the phantom used to assess quantitative accuracy. While the polychromatic attenuation image (A) shows typical beam-hardening artifacts, the basis material images (B,C) are unaffected of those and yield a clear decomposition into volume fractions of Ti and POM. The VMI at 80 keV (D) alongside with the effective atomic number (E) and electron density image (F) provide quantitative information of the studied object. The accuracy of the extracted quantities is evaluated in Table 2 and Fig 4. The depicted images correspond to the mean of 10 slices of the reconstructed image volume to improve the visual appearance of potential artifacts.

The reconstructed basis material image volumes (c. f. Fig 3B and 3C) were used to calculate images featuring the effective atomic number (c. f. Fig 3E) and electron density distribution (c. f. Fig 3F) using Eqs 9–13. The quantitative accuracy of the obtained voxel values was evaluated in cubical regions of 20x20x20 voxels in the center of each rod. The resultant mean values for the effective atomic number and the electron density are summarized in Table 2. The given error intervals correspond to the statistical uncertainty of the determined mean values (i.e. $\sigma /\sqrt{N}$, where *σ* is the standard deviation of the *N* voxels values within the evaluated region). The determined values show systematic deviations from the respective reference values. Thereby, the relative errors are within the range of 5 %. Given the high purity of the used materials (> 99.7%) the uncertainty of the reference values is negligible.

**Table 2:** Quantitative accuracy of the measured electron densities and effective atomic numbers in the material phantom. The reference values are according to Eqs 7 and 8. The error intervals correspond to the standard deviation of the mean value.

Material	$\frac{\rho_{\text{el},\text{ref}}}{10^{23}\;{\text{cm}}^{-3}}$	$\frac{\rho_{\text{el},\text{measuresd}}}{10^{23}\;{\text{cm}}^{-3}}$	$\frac{\text{Rel}.\text{error}}{\%}$	*Z*_eff,ref_	*Z*_eff,measured_	$\frac{\text{Rel}.\text{error}}{\%}$
Ti	12.56	12.80 ± 0.02	2.33	22.0	21.99 ± 0.01	-0.05
POM	4.52	4.55 ± 0.01	0.59	7.11	7.28 ± 0.03	2.38
Al	7.83	7.69 ± 0.01	-1.83	13.0	13.57 ± 0.01	4.12
PTFE	6.50	6.31 ± 0.01	-2.99	8.48	8.52 ± 0.02	0.72

**Fig 4:**
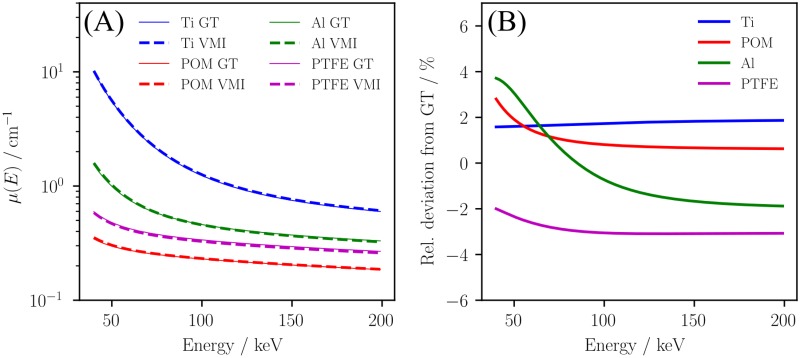
Quantitative accuray of virtual monoenergetic images. The VMIs calculated from the obtained basis material images (c. f. Fig 3B and 3C)) are compared to the theoretical attenuation values (label GT) of the studied materials (A) in the energy range from 40 to 200 keV. (B) shows the relative deviation between the values extracted from the measurement and the corresponding ground truth values. The theory values were taken from the XCOM database [31].

Fig 3D shows a virtual monoenergetic image at 80 keV, which was calculated from the basis material images using Eq 6. To study the quantitative accuracy of the obtained VMIs over a large energy range, the energy dependent attenuation coefficient was evaluated in each rod and compared to the materials’ repective ground truth values (c. f. Fig 4). Thereby, the energy dependent attenuation coefficients obtained in the measurements are in good agreement with the theory curves of the respective materials in the energy range from 40 to 200 keV (c. f. Fig 4A). Although, the materials that were not explicitly calibrated (Al and PTFE) show the highest errors, the relative deviation does not exceed a value of 4% over the whole evaluated energy range (c. f. Fig 4B).

**Fig 5:**
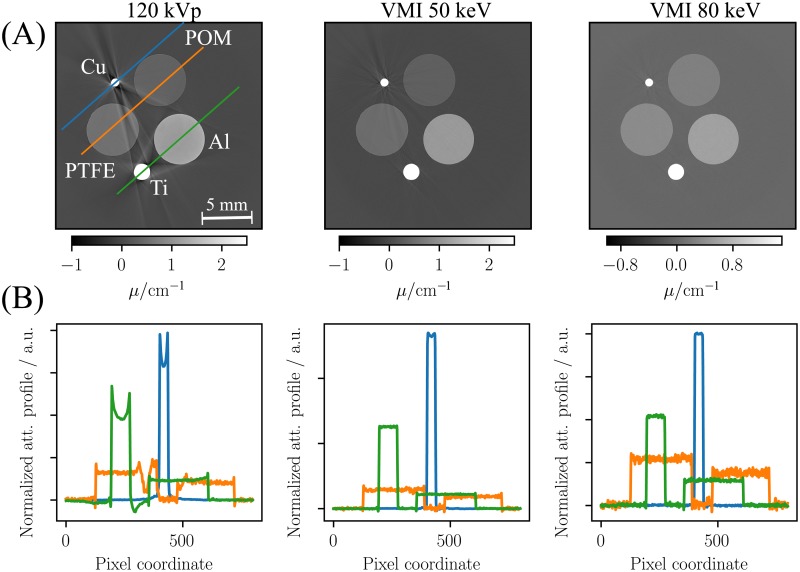
Spectral CT images of the phantom used to assess the capability of correcting beam-hardening. The upper row (A) shows the reconstructed image data after a copper wire was added to the phantom. The influence of the strongly attenuating copper wire was analyzed by line-plots (B). The depicted images correspond to the mean of 10 slices of the reconstructed image volume to improve the visual appearance of potential artifacts.

In order to study the method’s capability of handling beam-hardening properly a strongly attenuating copper wire (0.75 mm in diameter) was added to the evaluated phantom. Fig 5A shows the reconstructed image volumes of the corresponding CT scan. While the polychromatic attenuation image (120 kVp) exhibits severe streak artefacts arising due to beam-hardening, the VMIs at 50 and 80 keV are almost unaffected by the corresponding artefacts. Fig 5B shows lineplots through the different rods and reveals strong cupping in the titanium rod (green line) and the copper wire (blue line). In the VMIs the cupping is eliminated effectively, which indicates that beam hardening is modelled properly by the proposed forward model. While in the polychromatic attenuation image the line-profile of the teflon rod and the pom rod are strongly affected by beam hardening streaks (c.f. orange line), the line-profile is barely afflicted in the VMIs.

### 2.3 Proof-of-principle NDT applications

In order to demonstrate the benefits of spectral micro-CT over conventional micro-CT, we performed CT scans of two material science related samples. The tomography parameters of the scans are listed in Table 1.

The first object was a concrete drill core with a diameter of approximately 1.5 cm and primarily should serve for the illustration of the contrast enhancement achieved by the basis component decomposition. Prior to a dual-energy CT scan, we performed the calibration procedure as described in the materials and methods section of the paper.

**Fig 6:**
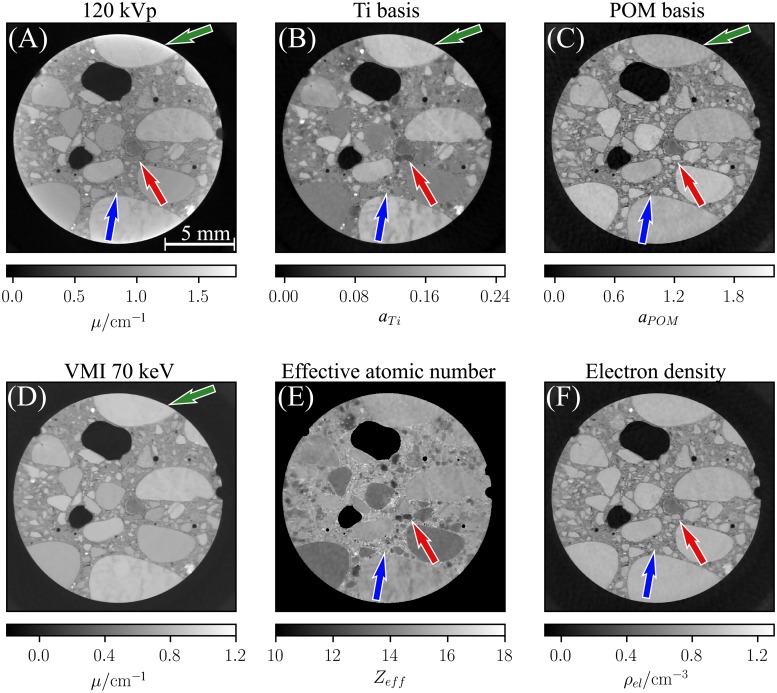
Conventional and spectral CT images of a concrete drill core. The conventional image (A) depicts the attenuation of the polychrmoatic spectrum, while the decomposed basis material images show the volume fractions of titanium (B) and POM (C). (D) gives the sample’s attenuation value at 70 keV. (E) and (F) depict the spatial distribution of the effective atomic number and the electron density within the sample, respectively.

Fig 6 shows an axial slice of the obtained images. Besides a conventional image depicting the attenuation of the polychromatic x-ray spectrum, the figure includes the basis material images. Compared to the conventional attenuation image (c. f. Fig 6A), the basis material images provide an enhanced image contrast. The red arrows in the images illustrate the enhanced contrast between different material inclusions, which is primarily caused by the strong dependency of the titanium basis (c. f. Fig 6B) on the atomic number. The enhanced contrast allows to better discriminate between different materials which is hardly possible in the polychromatic attenuation image. As the dependency of the POM basis (c. f. Fig 6C) on the atomic number is rather low, the POM image features only limited contrast between the material inclusions. However, the image yields a clearly visible segmentation between inclusions and cement, allowing for a better detectability of the fine granulation of the concrete (c.f. blue arrows Fig 6). As the effective atomic number image (c. f. Fig 6E) and the electron density image (c. f. Fig 6F) yield a direct representation of the sample’s chemical quantities, the above mentioned aspects are enhanced in these image modalities. At the edges of the drill core cupping artifacts are visible in the polychromatic attenuation image (green arrow), whereas these are eliminated in the spectral imaging results.

**Fig 7:**
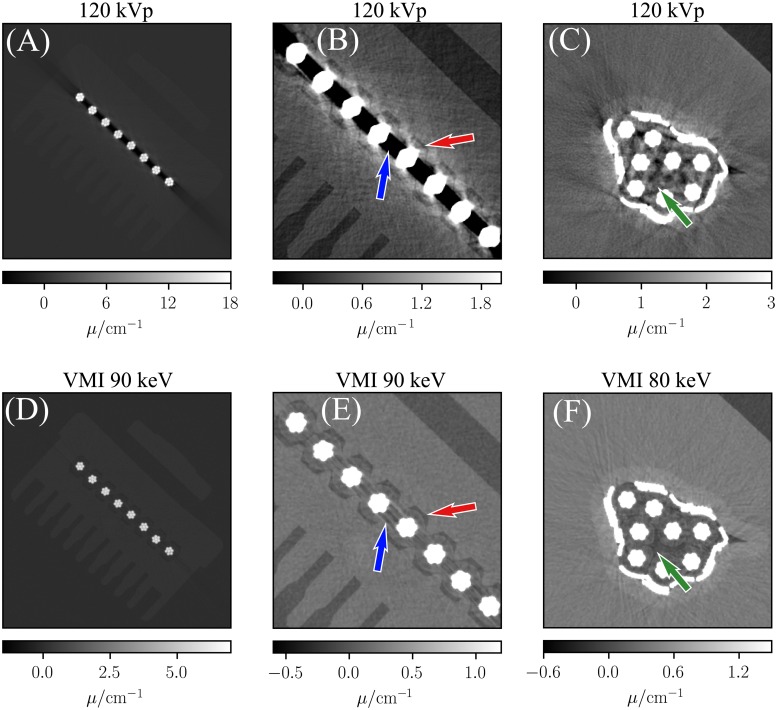
Conventional and spectral CT images of an ethernet connector. The top row depicts polychromatic attenuation images, while the bottom row shows VMIs of the corresponding sample regions. Subfigure (B) and (E) show more narrow windowed clippings of (A) and (D), respectively. (C) and (F) depict a different position inside the reconstructed volume. The energy levels of the shown VMIs were chosen in favor of best visual appearance.

As shown previously, the introduced method is able to model beam-hardening properly (c.f. Fig 5) and consequently promises superior performance compared to conventional imaging techniques. In order to study potential advantages concerning the reduction of beam-hardening artifacts, we performed a CT scan of an ethernet connector. Similar to the measurement of the drill core, the calibration procedure was performed. Fig 7 depicts conventional images of the sample acquired with a polychromatic spectrum at 120 kV peak voltage as well as corresponding VMIs calculated using Eq 6. The conventional images clearly suffers from beam-hardening artifacts. Thereby, the blue and red arrow in Fig 7B illustrate the effects of cupping and streak artifacts resulting in an obstruction of low-contrast features and reduced sharpness of edges. As indicated by the arrows in Fig 7E, the mentioned artifacts are strongly suppressed in the VMI image and the indicated features show enhanced quality. Although, the streak artifacts are not eliminated completely in the VMI (blue arrow), the isolation of the wires is still clearly visible and can be discriminated from surrounding plastic components (red arrow). Fig 7C and 7F show the cable bunch inside the ethernet connector. In the 120 kVp image streak artifacts obstruct the low contrast features, such as the isolation of the individual copper wire bunches. As highlighted by the green arrow, the streaks are strongly suppressed in the corresponding VMI and provide sufficient visibility of the mentioned low-contrast features.

## 3 Discussion

We evaluated the feasibility of spectral imaging for micro-CT applications in experimental measurements. The proposed method provides basis material images unaffected of beam-hardening and featuring quantitative image data. Different from other spectral imaging methods [15] the knowledge of system parameters is not required, but rather directly considered by a fast and simple calibration routine.

The reliability of the proposed calibration routine and the accuracy of the resultant decomposed basis material line-integrals was evaluated by the measurement of a test grid which consisted of pre-defined thickness combinations of titanium and POM. The relative bias between the decomposition results and the respective ground truth values are in the range of single digit percentage values (c. f. Fig 2) and therefore one order of magnitude higher than the results from numerical simulations in prior studies [16]. However, the observed deviations can be explained considering the additional effects mentioned in the introduction that are present in an experimental environment. Those were not taken into account in [16] and can affect the accuracy of the acquired data and therefore introduce an additional bias.

In phantom studies we evaluated the proposed method’s quantitative accuracy in computed tomography. Thereby, we exploited the basis material volume fractions obtained in the material decomposition to interplolate the chemical quantities of unkown materials from a set of basis materials used in the presented calibration routine. Our findings show a high accuracy for the determination of the effective atomic number and electron density values of unkown samples, even for high-Z materials (c. f. Table 2). The observed deviations are distinctly larger than the statistical uncertainties and hence are likely due to a systematic bias. As previously mentioned this bias can be induced by various effects, whereby in case of a CT scan instabilities of set-up components and ambient fluctuations become more dominant.

The obtained virtual monoenergetic images exhibit a good agreement with the reference values of the evaluated materials over a large energy range (c. f. Fig 4). As demonstrated in Fig 5 severe beam-hardening artefacts that are introduced by highly attenuating materials are efficiently reduced in VMIs.

The enhancement in contrast provided by the basis material images of the concrete drill core (c. f. Fig 6) provides advanced possibilities for the investigation of concrete samples. Thereby, the strong Z-dependency of the titanium basis offers new possibilities for the identification of aggregates and analysis of the composition of the concrete. The improved visibility of inclusions in the POM basis image might allow for better characterization of the grain size in the cement and improvements in the analysis of spacing factors within the concrete. In general, these characteristics in combination with the provided quantitative properties might be very beneficial for the microstructural analysis of conrete [37]. Apart from NDT applications, the additional information provided by the effective atomic number and the electron density image allows for the analysis of minerals in rock samples, which has previously been shown with a spectral imaging approach relying on an image-based calibration method [38]. However, image-based techniques mostly are inferior in terms of quantitative accuracy as beam-hardening can not be considered properly.

The reduction of beam-hardening artifacts in the spectral images of an ethernet connector is very promising for NDT of electronic components using x-rays. The safety demands of electronic connectors are becoming more and more important due to the trend of an increasing automatization in manufacturing processes and autonomous vehicles, where application-specific switches and sensors are critical devices. At the same time, the manufacturing of electronic connectors has become more complex as miniaturization has driven the need for components to become smaller with tighter tolerances, which requires a more accurate and detailed inspection. Therefore, micro-CT plays an increasingly important role in the quality assurance process, since it allows the inspection of both, the outer and the inner connector parts, with a very high precision and in an non-destructive manner. However, since electronic connectors contain high-absorbing materials such as copper wires and metal screws, the CT reconstruction suffers from strong beam-hardening, which makes a reliable inspection difficult or even impossible. Using a harder x-ray spectrum is typically associated with a decreased image contrast and the x-ray spectra emitted by tube sources need to be pre-filtered to eliminate low energies. This however will decrease the photon flux and thus leads to increased measurement times. As demonstrated in Fig 7, the proposed method provides an efficient suppression of beam-hardening artifacts along with preserving the image contrast sufficiently. This is particularly beneficial in NDT for the application of segmentation algorithms [39]. However, as shown in Fig 7E and 7F the streaks are not eliminated completely in the VMIs and minor artifacts remain in the depicted images. Whereas in general the energy-dependent attenuation of copper can be modelled by a linear combination of the attenuation coefficients of titanium and POM (c. f. Eq 2), this requires negatively signed POM line-integral values. As this can not be covered in the calibration routine, copper is far outside the calibrated line-integral space and therefore falsifies the decomposed line-integral values. A possible workaround for objects containing copper would be to use a more suitable calibration material than titanium. Thereby, copper itself or any material with a even higher atomic number would avoid the occurance of negative line-integral values when modeling the energy-dependent attenuation of copper.

Apart from that, scattered radiation can influence the decomposed line-integral values. Similar to conventional attenuation based-imaging, scattering will add a low frequency bias to the acquired projection data. However, the bias introduced to spectral bin data is in general magnified by material decomposition algorithms [40], because the inversion of the forward-model is a non-linear operation. In our studies the amount of scattered radiation is strongly mitigated by the small field-of-view and the small sample size. However, the scatter-to-primary ratio increases for strongly attenuating samples and hence also the influence of scattered radiation. Therefore, the remaining streaks in Fig 7E and 7F might at least be partially induced by scattered radiation.

## 4 Conclusion

In this paper we have shown that photon-counting spectral micro-CT is a feasible and viable alternative to conventional micro-CT. Spectral data acquisition is performed by state-of-the-art photon-counting detectors without increase of required measurement time. Instead, enhanced image quality and the availabilty of quantitative material properties provide new possibilities for material science and NDT applications.
